# Structural Insights into *E. coli* Porphobilinogen Deaminase during Synthesis and Exit of 1-Hydroxymethylbilane

**DOI:** 10.1371/journal.pcbi.1003484

**Published:** 2014-03-06

**Authors:** Navneet Bung, Meenakshi Pradhan, Harini Srinivasan, Gopalakrishnan Bulusu

**Affiliations:** TCS Innovation Labs-Hyderabad (Life Sciences Division), Tata Consultancy Services Limited, Hyderabad, India; Heidelberg Institute for Theoretical Studies (HITS gGmbH), Germany

## Abstract

Porphobilinogen deaminase (PBGD) catalyzes the formation of 1-hydroxymethylbilane (HMB), a crucial intermediate in tetrapyrrole biosynthesis, through a step-wise polymerization of four molecules of porphobilinogen (PBG), using a unique dipyrromethane (DPM) cofactor. Structural and biochemical studies have suggested residues with catalytic importance, but their specific role in the mechanism and the dynamic behavior of the protein with respect to the growing pyrrole chain remains unknown. Molecular dynamics simulations of the protein through the different stages of pyrrole chain elongation suggested that the compactness of the overall protein decreases progressively with addition of each pyrrole ring. Essential dynamics showed that domains move apart while the cofactor turn region moves towards the second domain, thus creating space for the pyrrole rings added at each stage. Residues of the flexible active site loop play a significant role in its modulation. Steered molecular dynamics was performed to predict the exit mechanism of HMB from PBGD at the end of the catalytic cycle. Based on the force profile and minimal structural changes the proposed path for the exit of HMB is through the space between the domains flanking the active site loop. Residues reported as catalytically important, also play an important role in the exit of HMB. Further, upon removal of HMB, the structure of PBGD gradually relaxes to resemble its initial stage structure, indicating its readiness to resume a new catalytic cycle.

## Author Summary

Heme is the prosthetic group at the core of the oxygen carrier metalloprotein hemoglobin. Heme consists of a tetrapyrrole called porphyrin bound to an iron ion. It is synthesized by the heme biosynthetic pathway, which is common to all eukaryotes and most prokaryotes. Porphobilinogen deaminase, an enzyme in the heme biosynthetic pathway, catalyzes the formation of a linear tetrapyrrole product, 1-hydroxymethylbilane, from four units of porphobilinogen. In this study we carried out molecular dynamics simulations to understand the structural changes that the enzyme undergoes while catalyzing this reaction. There are three segments to the study: 1) understanding the changes in the enzyme when the porphobilinogen units get attached to the dipyrromethane cofactor, thereby forming a polypyrrole chain; 2) exit of the product from the active site of the enzyme via steered molecular dynamics; and 3) the relaxation of the enzyme to the initial stage to resume its catalytic cycle. Molecular dynamics simulations of the protein through the different stages of pyrrole chain elongation gives insight into the motions of domains, active site loop and role of conserved active site residues in facilitating the accommodation of the polypyrrole chain. In addition to this, we propose a possible exit path for the product and demonstrate the relaxation of the enzyme after the exit of the product to resume the catalytic cycle.

## Introduction

**Figure 1:**
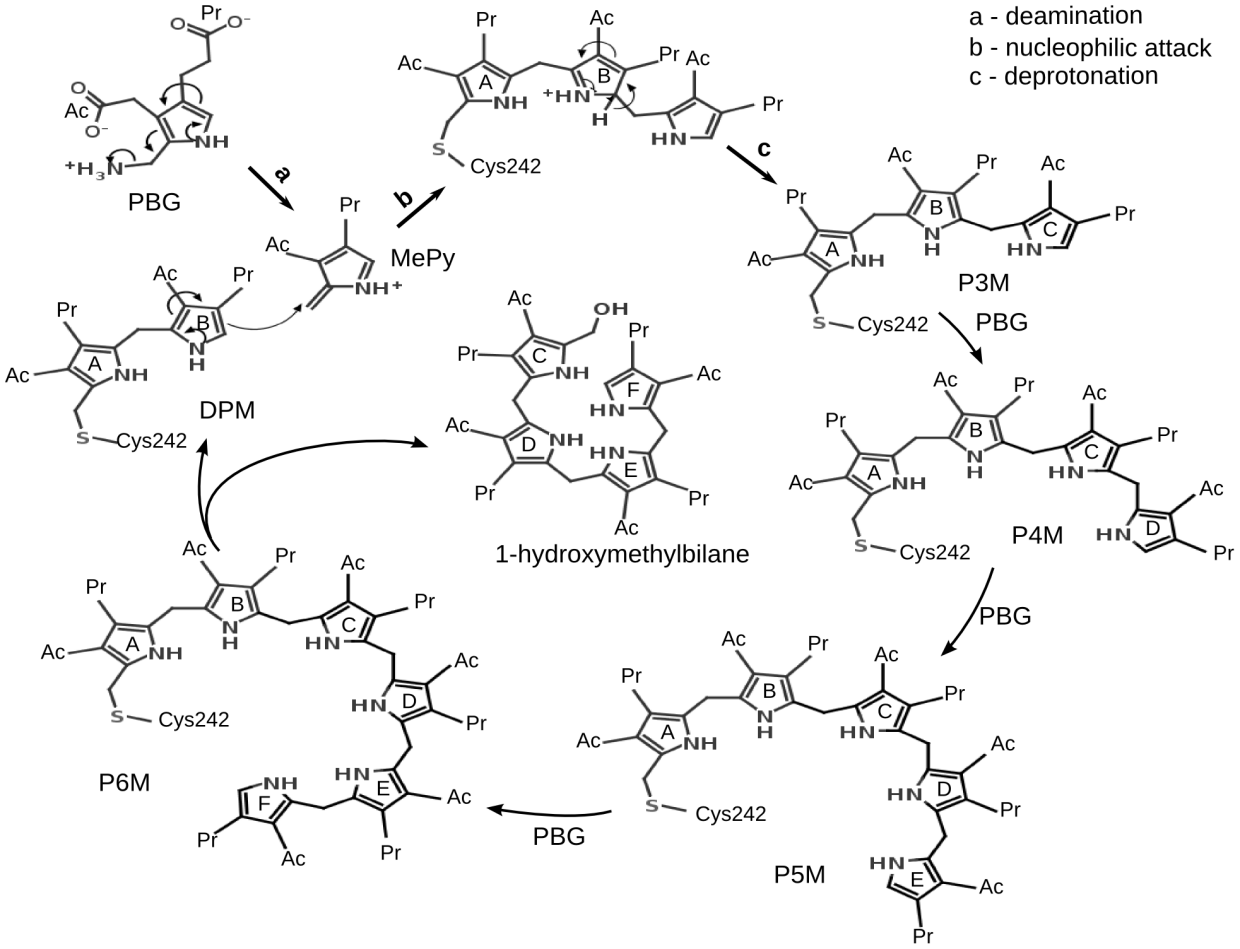
Mechanism of tetrapyrrole chain elongation catalyzed by PBGD. The scheme shows the deamination of porphobilinogen (PBG) to methylene pyrrolenine (MePy), the nucleophilic attack by the B ring of dipyrromethane (DPM) on MePy forming an intermediate that undergoes deprotonation to form a tripyrrole moiety (P3M). Subsequent additions of PBG elongates the chain to form tetrapyrrole (P4M), pentapyrrole (P5M) and hexapyrrole (P6M) moieties. In the last step, the tetrapyrrole product, 1-hydroxymethylbilane (HMB) is hydrolyzed from DPM. The rings of the elongating pyrrole chain are labeled as A, B, C, D, E and F starting from the pyrrole ring covalently attached to C242. The acetate and propionate side-groups of the pyrroles are denoted by -Ac and -Pr, respectively.

Heme, the second most abundant tetrapyrrole, serves as the cofactor for proteins involved in respiration and metabolism [1], [2]. Heme is synthesized through a well conserved and established heme biosynthetic pathway in all eukaryotes and most prokaryotes [3], [4]. Porphobilinogen deaminase (PBGD), an important enzyme in the pathway, is present in most of the organisms. PBGD (EC 2.5.1.61), a transferase, catalyzes the stepwise polymerization of four molecules of porphobilinogen (PBG) into a linear tetrapyrrole 1-hydroxymethylbilane (HMB), preuroporphyrinogen (Figure 1). PBGD is associated with acute intermittent porphyria, a hereditary autosomal dominant disorder, caused due to mutations in human PBGD (hPBGD) resulting in elevated levels of the heme precursors ALA (5-aminolevulinic acid) and PBG in the urine [5]. Dual functionality of PBGD has been reported in *Leptospira interrogans* [6] and has also been hypothesized in *Plasmodium falciparum* [7]. In these organisms, PBGD also cyclizes the usual linear preuroporphyrinogen product to uroporphyrinogen III. PBGD has a unique cofactor, dipyrromethane (DPM), which is covalently attached to a conserved cysteine through a thioether bond. This cofactor acts as a primer for the tetrapolymerization of PBG molecules [8]. The chain elongation takes place through repetition of a sequence of steps: (1) deamination of the incoming PBG substrate, (2) nucleophilic attack by the α-carbon atom of the terminal pyrrole ring of the enzyme-bound cofactor on the deaminated substrate and (3) deprotonation [8] (Figure 1).

**Figure 2:**
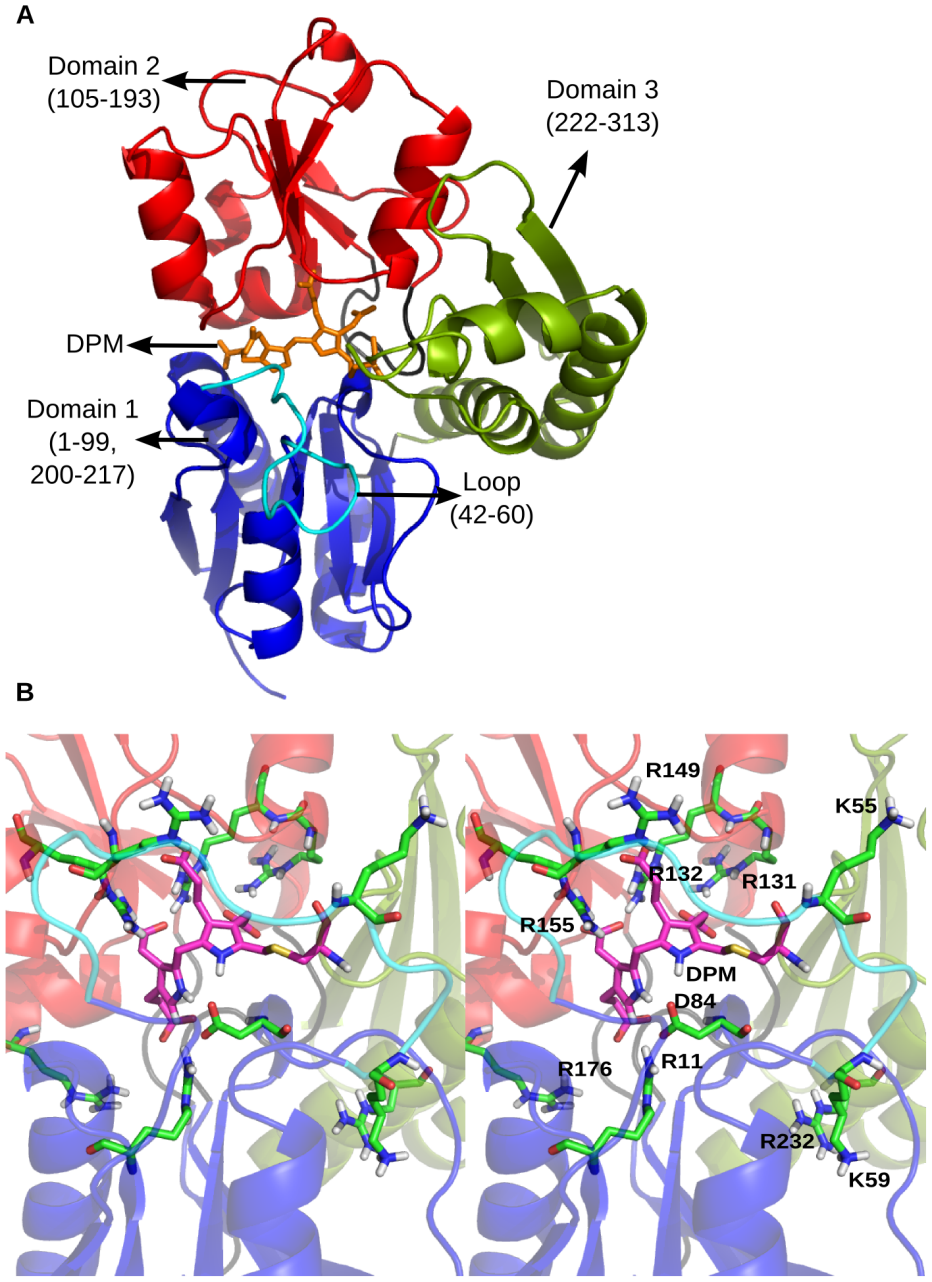
Loop modeled structure of *E.coli* PBGD and its conserved residues in the active site. A. The 3D coordinates of the *E.coli* PBGD were obtained from PDB (ID: 2YPN) and the missing loop region (42–60) was modeled using Modeller9v8. The same domain color scheme is followed throughout. B. Stereo view of the active site residues R11, D84, R131, R132, R149, R155, R176 and R232 along with conserved residues K55 and K59 (in green) and the DPM cofactor (in pink) shown as sticks.

Five crystal structures of *E. coli PBGD (*EcPBGD): 1GTK [9], 1YPN [10], 2YPN [11], 1AH5 [12], 1PDA [8]; two of hPBGD: 3ECR [13], 3EQ1 [5]; and most recently, one of *Arabidopsis thaliana* PBGD (AtPBGD): 4HTG [14] are available in the protein data bank (PDB) [15]. The structure of EcPBGD (Figure 2A) consists of 3 domains of α/β class. Domain 1 (1–99, 200–217) and domain 2 (105–193) share a topology similar to that of transferrins and periplasmic binding proteins [8], while domain 3 (222–313) differs having a three-stranded antiparallel β-sheet with one of its faces covered by three α-helices. The domains are connected by 3 hinge regions (100–104, 194–199, 218–221). The domain 3 interacts with domain 1, domain 2 and the inter-domain hinge regions primarily through polar interactions, though it also has a hydrophobic interface with domains 1 and 2 [8]. DPM is linked by a thioether bond to C242, on a flexible cofactor turn (residues 240–243) and lies in a cleft between domains 1 and 2 [16]. The coordinates for most of the residues in the flexible loop region (40–63), also known as ‘active site loop’ present in domain 1, were missing in all the available EcPBGD and hPBGD crystal structures [5], [16], [13] as they could not be determined. However, these were determined in the recent crystal structure of AtPBGD, 4HTG [14].

Structural and biochemical studies have indicated a single catalytic site in EcPBGD [8]. Mutational studies have suggested that several arginines (Figure 2B), conserved in PBGD across species, may be involved in the catalysis [17]. Mutations R11H, R149H, R155H, R176H and R232H have a detrimental effect on the activity of the enzyme [17]; while R131L and R132L affect the ability of protein to bind to DPM cofactor as the interaction of these arginines with the acetate (-Ac) and propionate (-Pr) side groups of the cofactor are lost [18], thus making the protein catalytically inactive. Also the mutations K55Q and K59Q affect the catalytic activity of the enzyme [19]. D84 has been suggested to play a key role in stabilizing the positive charges on the pyrrole rings during catalysis of chain elongation. D84E mutation shows a reduction in the enzyme's activity, whereas D84A and D84N mutations make the enzyme inactive [20].

Biochemical studies on hPBGD indicate that D99, R149, R167 and R173 (D84, R131, R149 and R155 respectively in EcPBGD) are involved in cofactor assembly and chain elongation [21]; D99 has also been suggested as a critical catalytic residue [13]. A homologous residue, D95, has been similarly implicated in AtPBGD [14]. Domain movement about the hinge regions has been predicted as facilitating the sequential entry of PBG molecules. Mutational studies on hPBGD, H120P, showed that the hinge residue, H120, is critical for the activity of the enzyme and its replacement by a proline leads to its inactivation [13].

*Plasmodium falciparum* PBGD (PfPBGD) is a larger protein than its *E. coli* and human homologues; whose structure is yet to be determined experimentally. PfPBGD has a leucine (L116) in place of an otherwise conserved lysine (K55 in EcPBGD). Nagaraj et al., have shown that L116K has higher activity than the wild type PfPBGD, producing both uroporphyrinogen I (non-enzymatic product) and uroporphyrinogen III, the product of URO3S, the next enzyme in the pathway [22]. *P. falciparum* is known to synthesize heme *de novo*, despite acquiring heme from the host red cell hemoglobin in the intraerythrocytic stage. Inhibition of its heme biosynthetic pathway leads to the death of the parasite [23], [24], emphasizing the importance of the enzyme for the parasite.

Louie et al., [16] have hypothesized two mechanisms for accommodating the elongating polypyrrole chain: (1) the sliding active site model in which the elongating chain is accommodated in the cavity within the protein and the domains adjust themselves to juxtapose the binding site and catalytic site residues near the terminal pyrrole ring; and (2) the moving chain model, in which the elongating chain is progressively pulled past the catalytic site placing the penultimate and terminal rings in the substrate binding site. Roberts et al., [14] hypothesized that the rotation of the bond linking the cofactor and conserved cysteine would cause the A and B rings to vacate their position for incoming pyrrole rings to bind at the same catalytic site, a process similar to the moving chain model. Experimental studies have helped to gain insights on the role of catalytically important residues of porphobilinogen deaminase.

All the hypotheses that have been proposed on the catalytic mechanism of PBGD were based on the structures of the DPM stage alone, as the structures of subsequent catalytic stages of PBGD are unknown. Consequently, the structural, conformational and domain dynamics of the various catalytic stages of PBGD and the role of individual amino acids in these processes are also not known. Hence, we have carried out extensive MD simulations to investigate the mechanism of the pyrrole chain elongation, its accommodation within the binding site, the concomitant domain motions and the role of the active site residues. Ligand entry and exit paths have always been of interest to structural biologists. We have studied the probable exit paths for the tetrapyrrole product (HMB) of PBGD, formed by hydrolysis of the hexapyrrole, using Steered Molecular Dynamics (SMD) [25]. We have also investigated relaxation of the protein after the exit of HMB. EcPBGD, the best characterized of all known PBGDs till date, has been used as the model system for this study.

## Results

### (I) Pyrrole chain elongation

**Figure 3:**
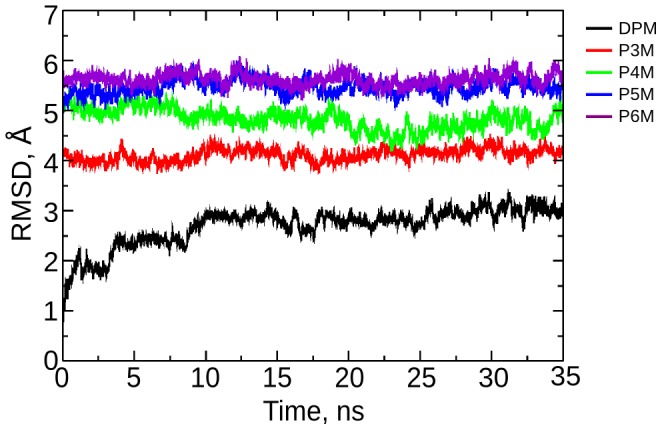
Root mean square deviation of the protein during pyrrole chain elongation. RMSD of the protein backbone, with respect to the 2YPN reference structure, at each stage of chain elongation.

**Figure 4:**
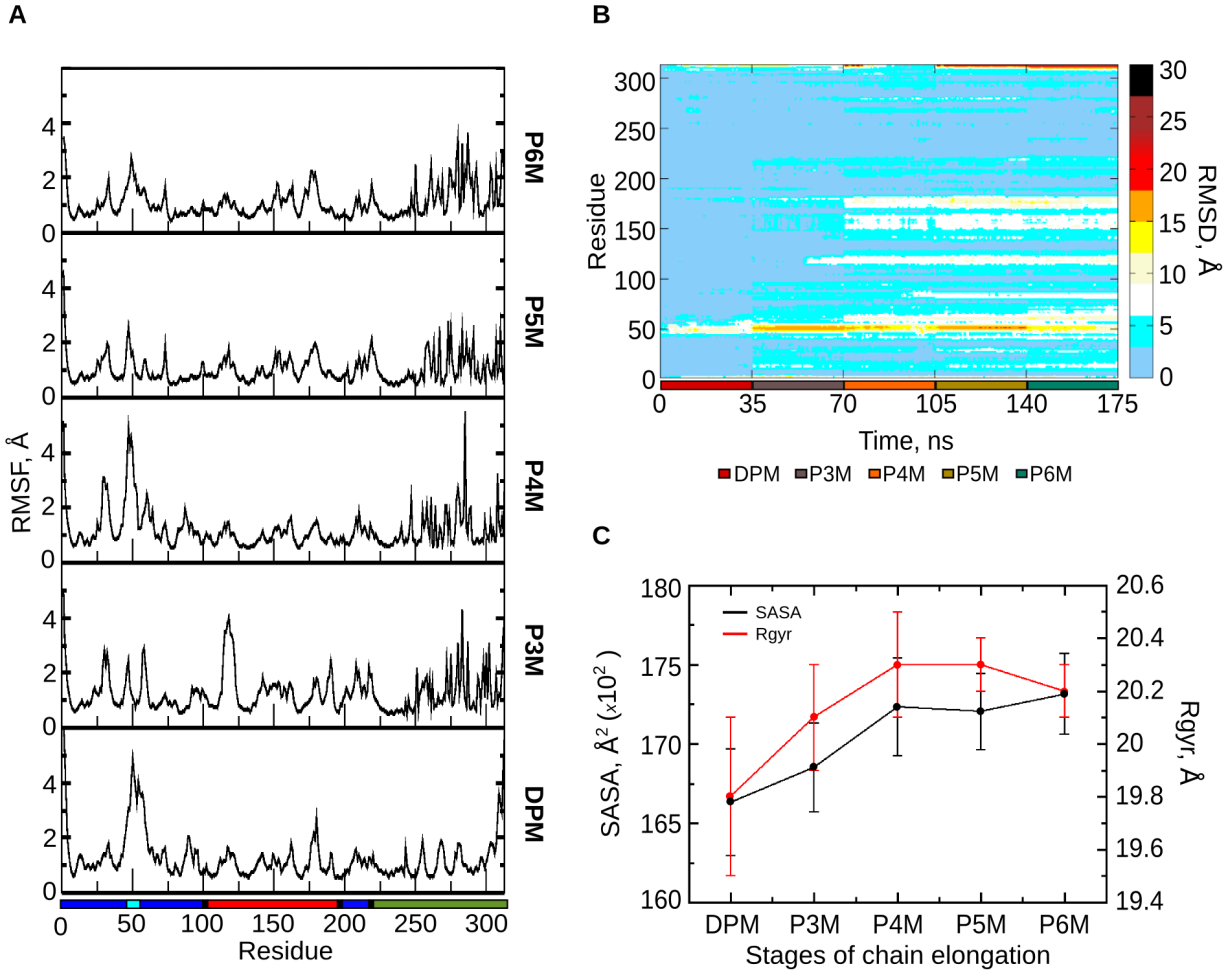
Protein dynamics during pyrrole chain elongation. A. RMSF plot of the protein from DPM to P6M stages. The color bar at the bottom corresponds to the domain demarcation (domain 1 – blue, domain 2 – red and domain 3 – green, active site loop – cyan, hinge regions – black). B. HeatMap showing the residue-wise contribution to RMSD of the protein through the different stages of simulation indicated by a color bar at the right. The simulation stages are denoted by a color bar along the abscissa. C. Solvent Accessible Surface Area (SASA) and Radius of gyration (Rgyr) values show the loss of compactness of the protein on addition of each PBG molecule through the stages of simulation from DPM to P6M. The error bars shown in the figure represent the standard deviation of the data from the mean.

The process of pyrrole chain elongation was studied by simulating PBGD at each of the four stages during the tetrapolymerization of PBG (after addition of each PBG unit) to understand the concomitant structural changes in the protein. The RMSD of the protein backbone at each stage of chain elongation relative to the EcPBGD reference structure is shown in Figure 3. The RMSD showed increase from DPM to P5M stage indicating structural changes upon addition of each porphobilinogen unit; the change in RMSD, however, is negligible going from P5M to P6M stage. In Figure 4B, the color code of the HeatMap explains the residue-wise RMSD contribution. The active site loop residues (42–60) and the domain 2 region contribute significantly to the observed structural deviations. High RMSD is observed for the active site loop from DPM stage and for domain 2 from P4M stage onwards. The RMSF plot in Figure 4A, showed that, in DPM and P4M stages, the fluctuation in the active site loop region is high, while that in the domain 2 is low. The opposite is observed in the P3M stage, where the fluctuation is high in parts of domain 2 and low for the active site loop. Fluctuations of ∼2 Å were observed in the P5M and P6M stages, which are small compared to that in other stages. These observations indicate that in order to accommodate the growing pyrrole chain, either the active site loop or domain 2 readjust to widen the active site cleft. SASA and Rgyr values (Figure 4C) are also in accordance with the above observations indicating that after the P4M stage, the entire pyrrole chain gets accommodated within the expanded active site with minimal structural changes in the protein.

#### Active site loop dynamics

**Figure 5:**
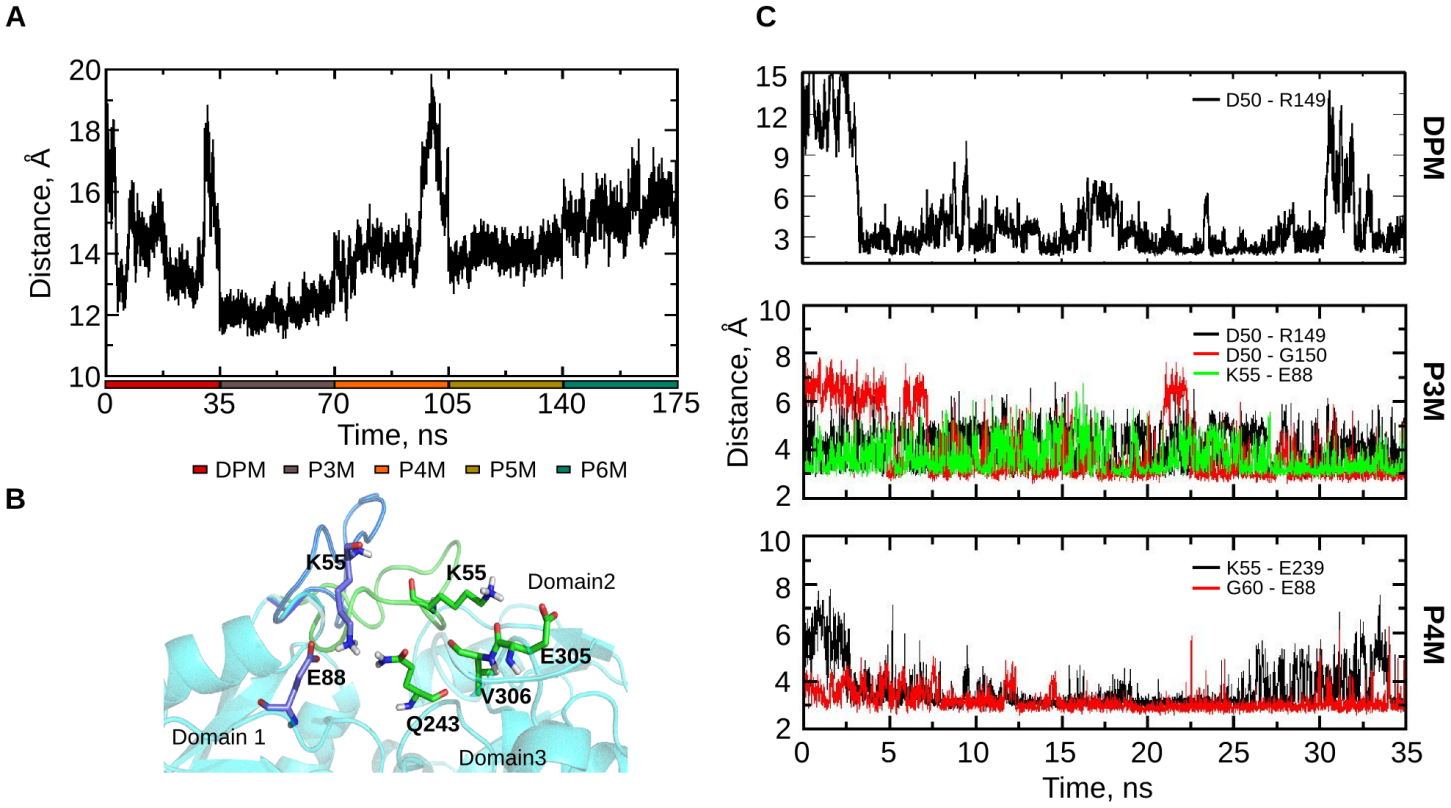
Loop dynamics during pyrrole chain elongation. A. Plot of distance between centers of mass of the loop residues (42–60) and the active site residues (11, 19, 84, 131, 132, 155, 176, 242) to track the loop movement in the different stages of the simulation. B. Interaction of K55 with E88 (open loop conformation denoted in blue color) and with V306, E305 and Q243 (closed loop conformation denoted in green color) regulate the loop movement in DPM stage. C. Distance graphs depicting the interaction of D50 with R149 during DPM stage to regulate loop movement (along with K55 interactions); interaction of D50 with R149 (black), D50 with G150 (red) and K55 with E88 (green), involved in loop movement during the P3M stage; Interaction of K55 with E239 (black) and G60 with E88 (red) involved in loop movement during the P4M stage.

The dynamics of the active site loop in conjunction with the active site residues was tracked for all stages of PBG polymerization (Figure 5A). The loop fluctuates during the DPM stage, remains near the active site in the P3M stage and then moves away from the active site in P4M to P6M stages. The active site loop is seen to move back and forth from the active site cleft in the DPM stage. During this movement, loop residue D50 interacts with R149 and K55 interacts with Q243, E305 and V306 with an occupancy of 41% along the trajectory. As a result of these interactions, the loop remains close to the active site (closed conformation of the loop). When the loop stays away from the active site (open conformation of the loop), K55 is observed to be interacting with E88 (Figure 5B). In the P3M stage, the loop is seen to remain close to the active site. D50 interacts with R149 throughout the trajectory, while K55 interacts with E88 (Figure 5C); D50 also interacts with the backbone atoms of G150. In the P4M stage, the loop starts moving away from the active site, which leads to replacement of the interaction of D50 with R149 by the interaction of G60 with E88 (Figure 5C). Also, K55 forms a salt-bridge with E239. No interactions are observed between the loop residues and the active site residues in P5M and P6M stages as the loop stays away from the active site, with K55 interacting with the backbone of C-terminal residue N308, forming a β-bridge. Louie et al., proposed that the active site loop plays an important role during the enzyme catalysis [16]. The conformational dynamics of the loop guided by strong interactions is an indication of its possible role in the enzyme catalysis.

#### Structural changes in the protein during the chain elongation process

**Figure 6:**
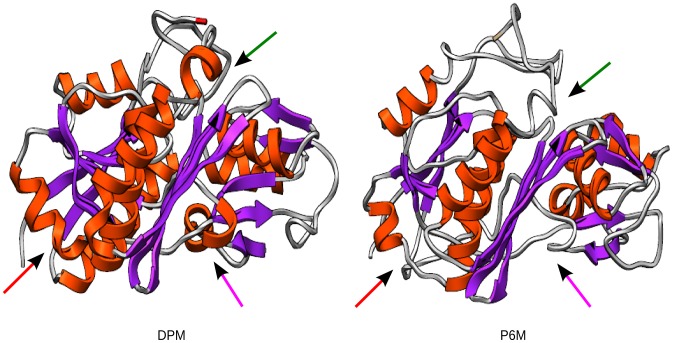
Structural changes in PBGD during DPM to P6M stages. Structural changes observed in the protein in DPM and P6M stages show the length of beta sheets and helices in domain 1 shortens (red arrow) in P6M stage, a shorter helix is observed in domain 3 (green arrow) and the hinge region between domain 1 and domain 2 uncoils (pink arrow) in the P6M stage.

Comparison of the average structures of PBGD from the initial DPM stage and the final P6M stage showed noticeable differences in their secondary structures. As seen in Figure 6, the length of β-sheets and helices in domain 1 have become shorter in the P6M stage compared to the DPM stage. A helix in domain 3 (P280-N296) gets shortened and a short helix in the hinge region joining domain 1 and domain 2 (around L193) gets uncoiled. These structural changes are a result of widening the gap between the domains 1 and 2 during the polypyrrole chain elongation.

#### Combined dynamics of the protein during tetrapyrrole chain elongation

**Figure 7:**
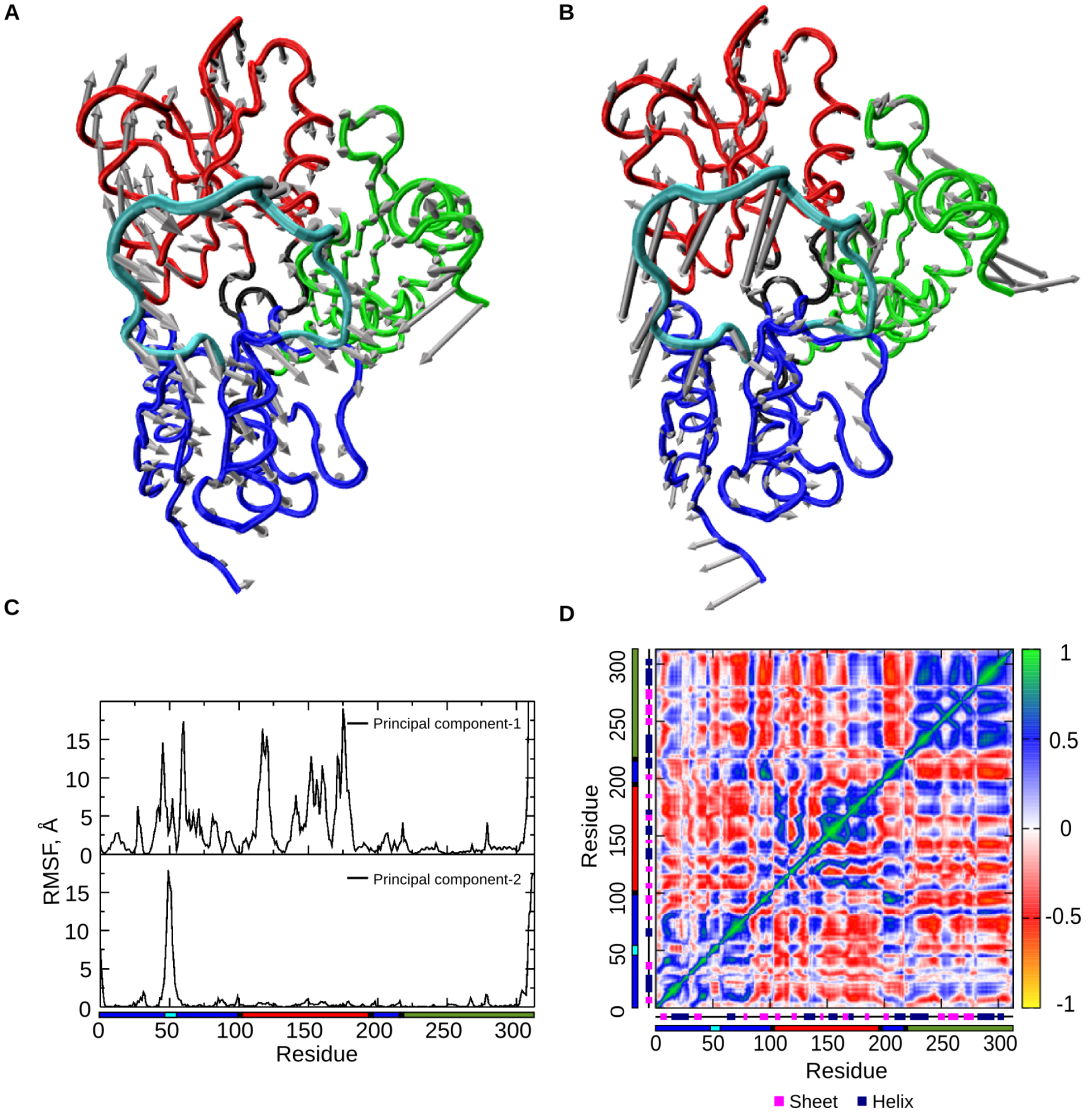
Combined essential dynamics and DCCM of the concatenated trajectory. A. Principal component 1 (Video S1) and B. Principal component 2 (Video S2) of the combined trajectory analysis from DPM to P6M stages. C. RMSF corresponding to the principal component 1 and 2 with domain demarcation along the abscissa. D. Dynamic Cross Correlation Map on the concatenated trajectory of all stages of simulation showing the correlation between the domain movements with the color bar at right indicating the intensity of correlation. Domain demarcations and the secondary structure elements (helix and sheet) are also shown along the axes.

Cumulative effect of the domain movements observed during the individual steps of the chain elongation process has been studied by concatenating the trajectories of all stages (from DPM to P6M). Principal component analysis (PCA), also known as essential dynamics was performed on the combined trajectory; the first two principal components captured around 70% of all protein motions. The principal component 1 (Figure 7A, 7C, Video S1) indicated domain 1 to be negatively correlated to domain 2. Parts of domain 1 are positively correlated to regions of domain 3, while the active site loop region along with β5_1_ strand (5th strand in domain 1, residues 200–206) and α4_1_ helix (4th helix in domain 1, residues 210–217), are negatively correlated to domain 3. The movement of the cofactor turn was observed to be towards domain 2 with the active site loop moving away synchronously. This indicates that with the growing pyrrole chain, the cofactor turn moves into the active site cleft, inclined towards domain 2. The principal component 2 also indicated similar correlations between domains with major fluctuations in the active site loop (Figure 7B, 7C, Video S2) indicating it to be negatively correlated to domain 2.

The DCCM plot (Figure 7D) of the concatenated trajectory also supports the observations made from PCA. The plot showed that domain 1 and domain 2 are negatively correlated to each other. Domain 3 (222–313) is seen to be positively correlated to residues 1–45 and 80–90 of domain 1 and negatively correlated to residues 46–80, 90–100 and 200–217 of domain 1. Also, domain 3 (222–313) is negatively correlated with the region 105–169 in domain 2 while it is positively correlated with the region 170–199 in domain 2.

#### Volume of active site cavity during tetrapyrrole elongation

**Figure 8:**
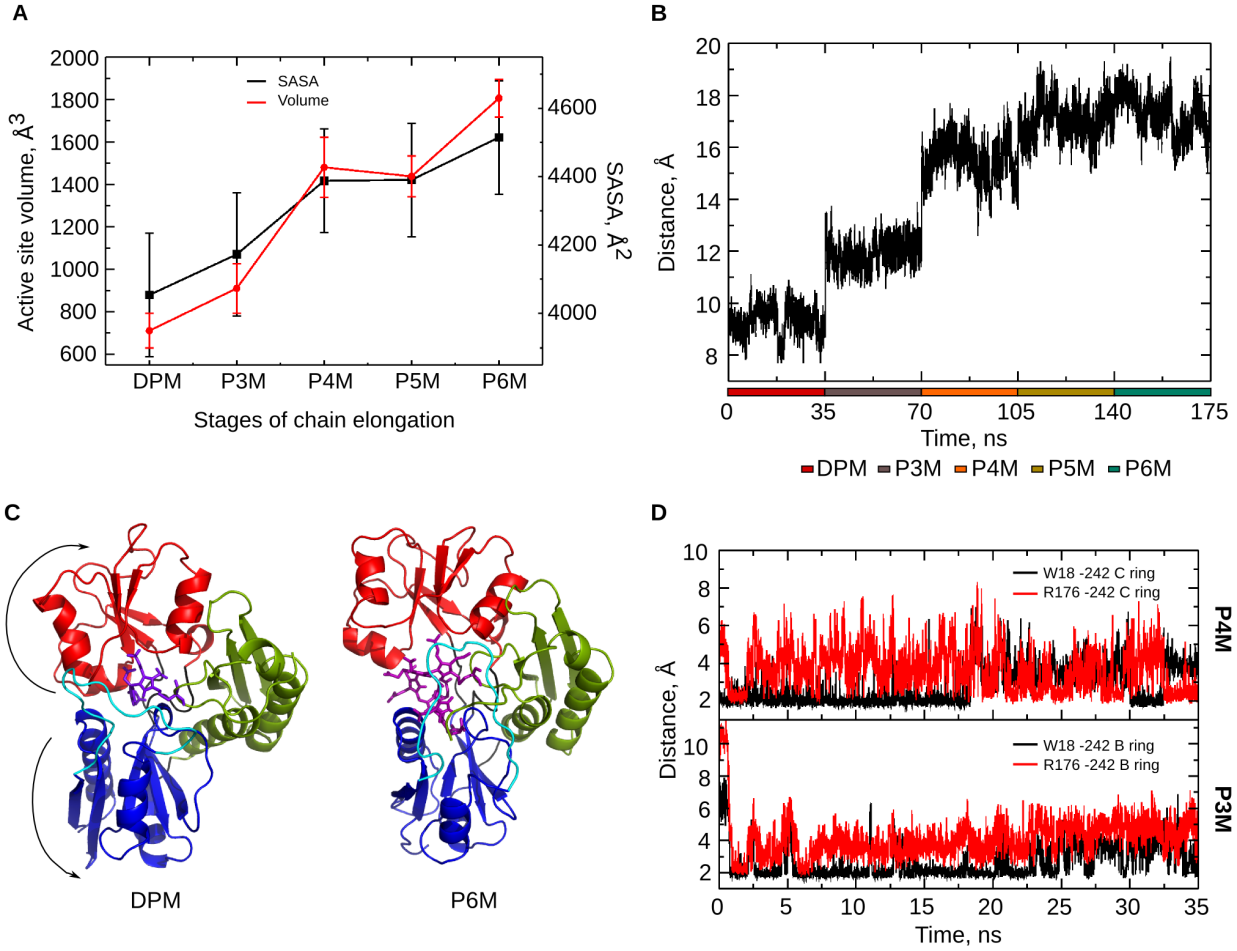
Polypyrrole chain accommodation. A. Volume of the active site and cumulative SASA of the active site residues (as reported in Table 1) show an increase from DPM to P6M stage with the addition of each PBG molecule. B. Graph showing the increase in domain separation between domain 1 and 2 during the catalytic stages of PBGD. C. Polypyrrole accommodation within the active site cleft as a result of major domain movements during chain elongation; snapshots of only DPM & P6M stages are shown. D. Graphs of the interaction between W18 and R176 with B ring of pyrrole chain in P3M stage and with C ring of pyrrole chain in P4M stage showing the shift in interactions to accommodate the polypyrrole chain.

Volume analysis of the active site cavity showed that it increases from DPM to P6M stage contributed mainly by the active site loop and the domain movements (Figure 8A). The active site loop remained in a closed conformation in the P3M stage and the distance between domain 1 and 2 increased from 9.5 Å in DPM stage to 12 Å with concomitant expansion of the active site volume to accommodate the third pyrrole unit (Figure 8B). In the P4M stage, the loop moved away from the active site cavity (Figure 5A); moreover, the distance between domain 1 and domain 2 increased to 16 Å (Figure 8B). This resulted in an increase in the active site volume to 1497±140 Å^3^ (Figure 8A) that is relatively large compared to that observed in the P3M stage, 910±110 Å^3^. Also, the space created for the polypyrrole chain in P4M stage is sufficient to accommodate another pyrrole unit in the P5M stage with minimal rearrangement of side chains of surrounding residues. Thus, the change in volume is negligible going from P4M to P5M stage. In the P6M stage, the active site loop moved further away from the cavity (Figure 5A), thereby, accommodating another pyrrole unit and creating sufficient space for the exit of product, HMB. These results are in accordance with the cumulative SASA values of the residues in the active site that interact with polypyrrole during the stages of chain elongation (Figure 8A).

#### Accommodation of the growing pyrrole chain

The dynamics of the growing pyrrole chain and its accommodation within the active site (Figure 8C) is best understood by studying the interactions between the growing chain and surrounding protein residues (Table 1, Figure S1). From Figure 8D, it is observed that there is a shift in the interactions of W18 and R176 from the B ring in P3M to the C ring in P4M during chain elongation. This pulls the polypyrrole in the P4M stage adjusting it in a way that the chain inclines towards domain 2 [26], allowing space for the additional pyrrole rings in P5M and P6M stages. The polypyrrole chain gets accommodated within the active site in a completely curled conformation. The pyrrole rings interact with most of the active site residues in domain 1 and 2 during all stages of elongation (Table 1). The terminal pyrrole ring interacts with similar set of residues in each of the stages (Table 1), thus providing a suitable environment for catalytic action on the incoming PBG molecule.

**Table 1:** Amino acids in PBGD interacting with the polypyrrole chain during each stage of chain elongation.

Stages	Residues interacting with the elongating pyrrole chain	Residues interacting with the terminal ring in each stage
DPM	S81, K83, D84, T127, S128, S129, R132, R155, L169, A170*, Q198 and G199*	S81, K83, D84, R132, R155, L169*, A170*, Q198 and G199*
P3M	W18, Q19, P52, S81, M82*, K83, D84, S128, S129, R131, R132, R155, A170*, G173, Q198 and G199*	Q19, S81, M82*, D84, Q198 and G199*
P4M	R11, L15*, A16*, W18, Q19, S81, M82*, K83*, D84*, S128, S129, R131, R132, R155, A170*, V171*, A172*, R176, G173* and Q198	R11, L15*, A16*, Q19, S81, M82*, K83* and D84*
P5M	R11, L15*, W18, Q19, T51*, P52*, H80, S81, K83, D84*, V85*, S128, S129, R131, R132, N151, R155, V171*, A172*, R176, Q198 and G199*	R11, Q19, H80, K83, D84* and V85*
P6M	R11, S13, W18, Q19, S81, K83, D84*, V85*, S129, R131, R149, R155, V171*, A172*, R176 and Q198	R11, S13, S81, K83, D84*, V85* and R149

* denotes interaction with backbone atoms of the residue.

Residues interacting with the pyrrole chain and its terminal ring at each stage of chain elongation.

The terminal ring is observed to be interacting with either R11 or Q19 (Table 1) from P3M stage onwards, which supported the hypothesis of Song et al., who suggested involvement of R11 and Q19 in the deamination of the incoming PBG units [13]. The terminal pyrrole ring is expected to be located close to the entry point of PBG units and proximal to R11 or Q19, which could facilitate the deamination process of incoming PBG units.

#### Role of active site residues in the catalytic mechanism

**Figure 9:**
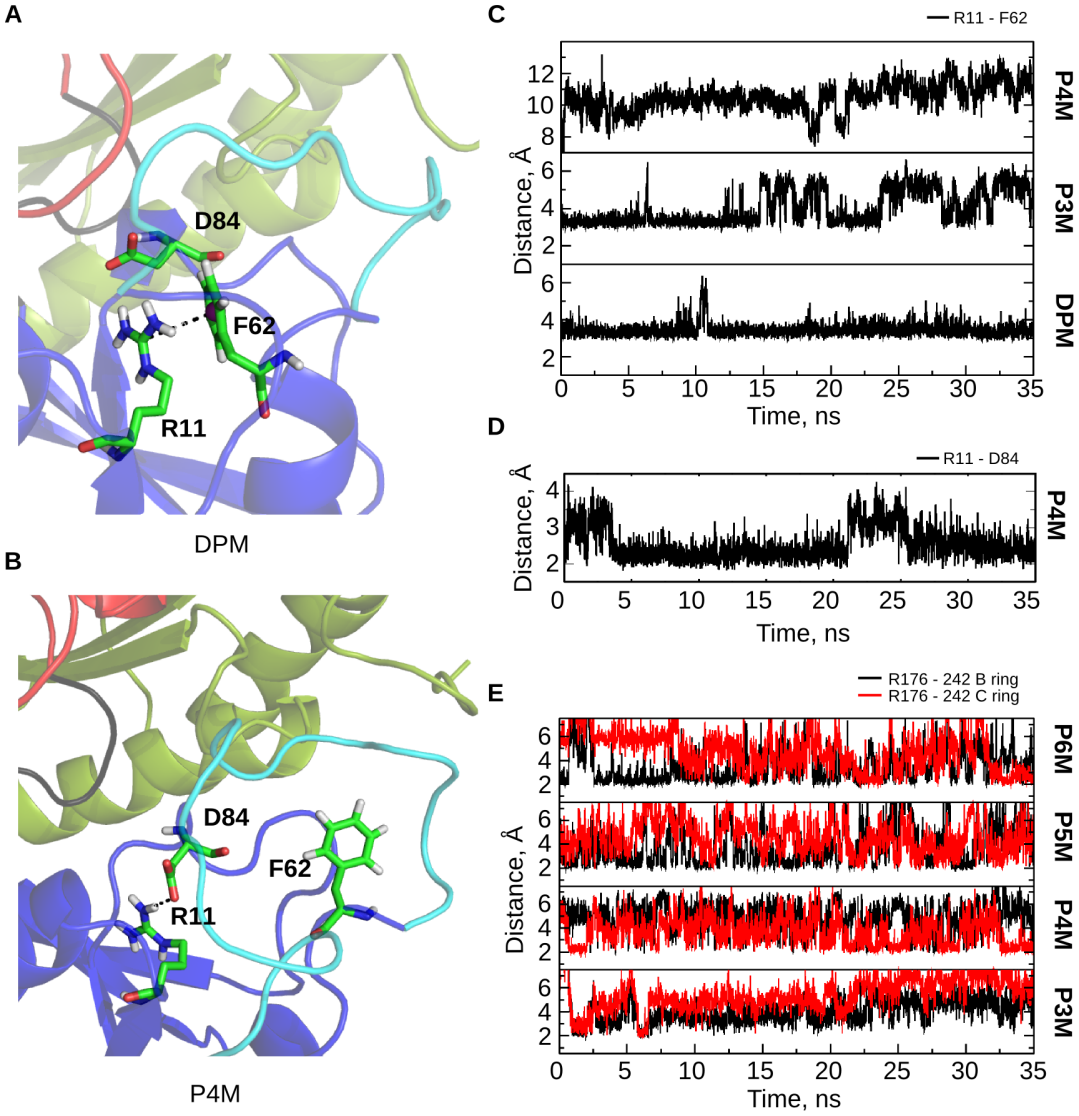
Interactions of R11, F62, D84 and R176. A. A closer view of the stacking interaction of R11 with F62 present at the base of the active site loop measured by the distance between the CZ atom of R11 and center of mass of the phenyl ring of F62 in the DPM stage. B. In the P4M stage D84 interacts with R11, disrupting the stacking of R11 with F62. C. Stacking interaction of R11 with F62 keeps the active site loop in a position facilitating its movement during DPM and P3M stages, shown as a distance graph between the CZ atom of R11 and the center of mass of the phenyl ring of F62 in DPM, P3M and P4M stages. D. Distance graph depicting the interaction of R11 with D84 in the P4M stage which causes the stacking between R11 and F62 to break. E. Distance graphs depicting the interaction of R176 with the B (in black) and C (in red) rings of the polypyrrole chain during the stages of chain elongation.

Active site residues R11, D84 and R176 appear to be involved in controlling crucial steps during tetrapyrrole formation. In the DPM stage (E stage, as per Jordan et al., nomenclature [17]), R11 stacks with F62, present at the base of the active site loop (Figure 9A), throughout the trajectory (Figure 9C), while D50 interacts with R149 and K55 interacts with Q243, E305 and V306 (Figure 5B, 5C) to regulate the loop movement.

In the P3M stage (ES stage), the loop is seen to remain close to the active site; D50 interacts with both R149 and G150 (Figure 5C), while K55 interacts with E88. The stacking of R11 continues with F62 (Figure 9C) juxtaposing the active site loop near the active site, which may be aiding catalysis during the initial stages of pyrrole chain elongation. This is supported by biochemical studies: R11H affects the E to ES stage and therefore the binding and attachment of first substrate, resulting in no ES complex formed [17].

In the P4M stage (ES2 stage), R11 interacts with D84 throughout the trajectory (Figure 9D). As the pyrrole chain elongates, D84 starts interacting with R11 thus, breaking the stacking of R11 with F62 that was persistent till the P3M stage (Figure 9B), causing the flexible loop to slide away from the active site. Therefore, in the absence of D84, stacking of F62 with R11 would have continued to keep the loop close to the active site, possibly obstructing further catalysis. Mutations D84A and D84N caused the enzyme to stall in P4M stage (ES2) [20], thus supporting its role in catalysis. Song et al., suggested that D99 in human PBGD (analogous to D84 in EcPBGD) could play a role in the nucleophilic attack and in the deprotonation after the formation of the new C-C bond between the polypyrrole chain and the incoming pyrrole ring [13]. They also hypothesized that other residues in the vicinity, R26, Q34 and R195, could play a role in the protonation of the incoming PBG molecule. Roberts et al., hypothesized that the aspartate D95 in AtPBGD (analogous to D84 in EcPBGD) takes part in all the catalytic steps [14]. D95 in AtPBGD is implicated to take part in deamination and nucleophilic attack. From the present study, we suggest that positively charged residues which lie close to the elongating pyrrole chain could take part in protonation of the incoming PBG molecule, while negatively charged residues could assist in the deamination and nucleophilic attack.

In the P3M stage (ES), R176 has a pulling effect on the B ring of the pyrrole chain (Figure 9E). This effect continues in the P4M stage (ES2) with interaction of the B and C rings of the pyrrole chain with R176 throughout the trajectory, aiding in accommodation of the pyrrole chain during elongation. The inclination of pyrrole chain towards domain 2 continues in the P5M (ES3) and P6M (ES4) stages as well. We hypothesize that in the absence of R176 there is no pulling effect on the pyrrole chain towards domain 2 and this may cause steric hindrance for the incoming PBG units during chain elongation. This is in accordance with the biochemical studies R176H mutation that affects the progress of ES (P3M) to ES2 (P4M) and ES2 (P4M) to ES3 (P5M) stages [17].

### (II) Exit mechanism

**Figure 10:**
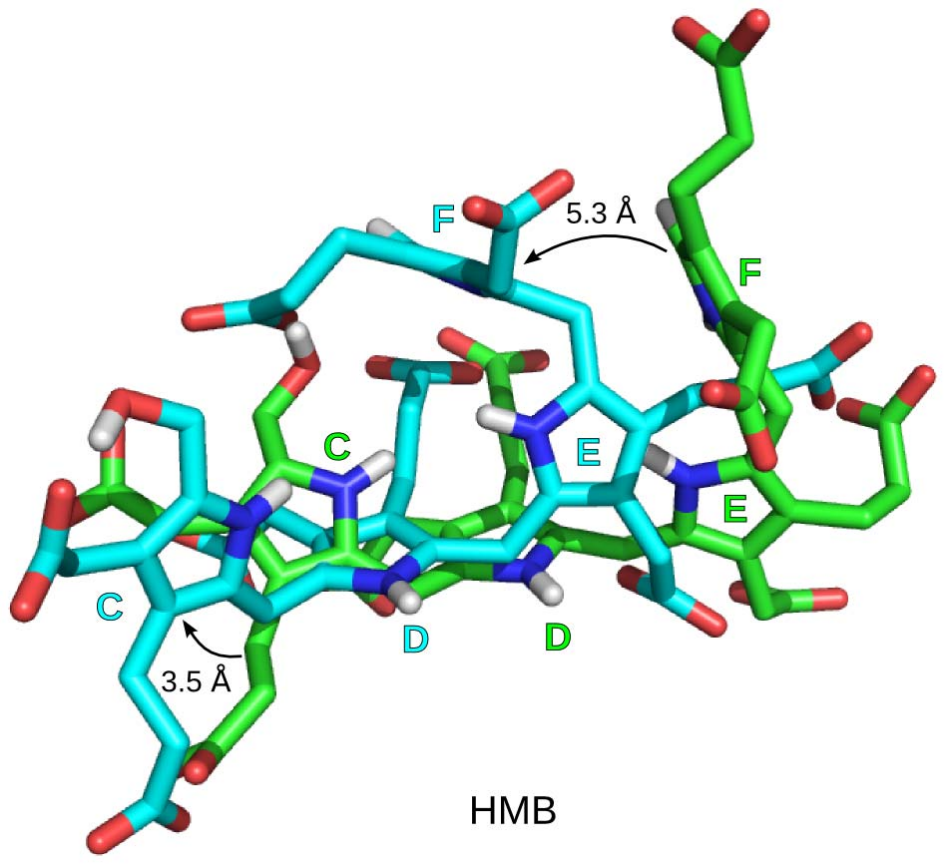
Conformation of 1-hydroxymethylbilane during the HMB stage simulation. With reference to initial conformation of HMB (green) the C and F rings are displaced by a distance of 3.5 Å and 5.3 Å respectively in the structure at the end of simulation (cyan) during the HMB stage.

**Figure 11:**
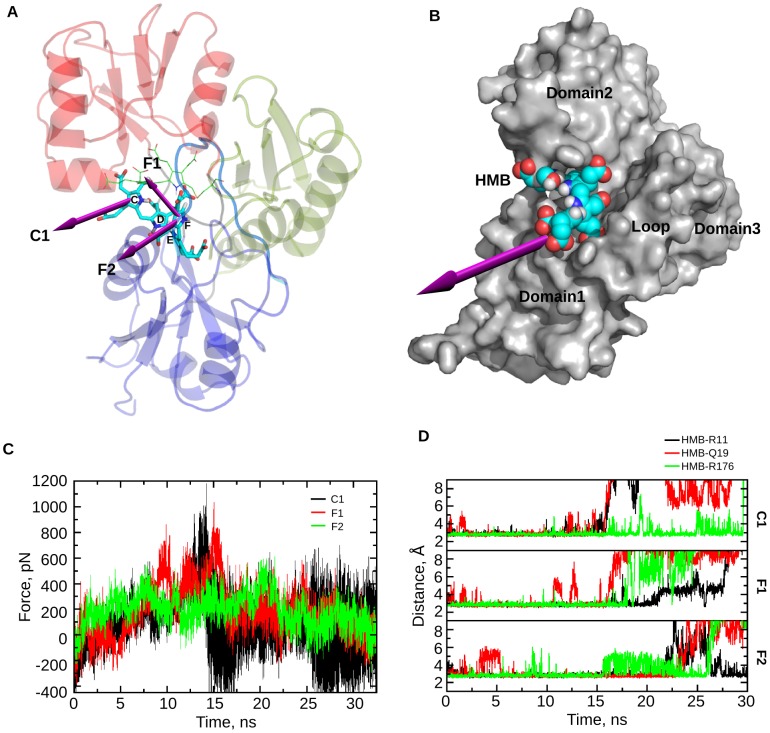
Exit mechanism of HMB from PBGD. A. Structure of PBGD showing probable exit directions, either from C or F ring of the HMB unit, that are considered for SMD simulations: C1 (Direction from the center of mass of the C ring in HMB towards the interface of domain 1 and domain 2), F1 (Direction from the center of mass of the F ring in HMB towards the active site loop), F2 (Direction from the center of mass of the F ring in HMB towards the interface between the active site loop and domain 1). B. Surface representation of the structure of PBGD showing the most probable path predicted for the exit of HMB through the space between domain 1, domain 2 and the active site loop (Video S3). C. Force as a function of time during the SMD runs in 3 different exit paths: C1, F1 and F2. D. Graphs showing the interactions of R11, Q19 and R176 with HMB during the SMD runs through C1, F1, and F2 path, indicating the possible role of these catalytically important residues in the exit of the product.

After studying the dynamics of PBGD during tetrapolymerization process, the hexapyrrole was cleaved forming the tetrapyrrole product, HMB, leaving DPM cofactor attached to PBGD (HMB stage). The HMB stage was simulated to study the exit mechanism of the product, HMB from the protein. During the simulation it was observed that the C ring of HMB slightly moved towards the opening between domain 1 and domain 2 and the F ring moved towards the space formed by the loop and domain 2 (Figure 10). As it would require longer simulation time for the product to exit from the PBGD, an external force was used to overcome the energy barriers. CAVER was used to detect channels for the exit of product (Figure S2). Based on the result from the HMB stage and CAVER, SMD was performed to study the exit of HMB from PBGD. Several trial runs, with varying pulling rate, as well as the final SMD runs were tried along directions of C1, F1 and F2 (Figure 11A). The magnitude of SMD expulsion forces is comparable to the unbinding forces of other protein-ligand systems [27].

In SMD along C1 path, the E, C and D rings of HMB interact with R11, Y22 and Q198 respectively until about 11 ns. An increase in the pull force is observed around 13 ns when these interactions start breaking (Figure 11C). The loss of interactions of the C ring of HMB moiety with R176, and the side chains of the E ring with K83, D84, V85 and Q19 cause the fluctuations in force profile till the product exits around 26 ns.

In SMD along F1 path, the pull force peaks are observed around 11 ns and 15 ns (Figure 11C). Loss of electrostatic interactions of the E ring of HMB with S81, D84 and V85 and the D ring of HMB with Q198 occur during the peak at 11ns as the product starts to exit. The peak around 15 ns corresponds to the loss of interactions of the C ring of HMB with N151 and R176 and the E ring with R11, Q19 and K83.

SMD through F2 path has the lowest force profile (Figure 11C); force peaks are observed around 8ns, 15ns and 20ns. The first peak corresponds to the alignment of the F ring of tetrapyrrole chain perpendicular to the α1_1_ helix in domain 1, while the second peak, around 15 ns, corresponds to the loss of interaction of the C ring with R176. The last peak around 20 ns corresponds to the interactions of the C ring with W18, Y22, K175, the D and C ring with Q19, the E ring with R11 and the E, D and C rings with K83 as they start breaking one after another, till the exit of HMB moiety from the protein at around 32 ns.

R11, Q19, K83, R176 are involved in each of the exit paths considered (Table 2), of which R11, Q19 and R176 have been suggested to be involved in the catalytic mechanism of the enzyme. Atleast one of these residues (R11, Q19 and R176) interacts with HMB till its exit from PBGD (Figure 11D), indicating their potential role in the exit of the product. Based on the SMD analysis, the favorable path for the product exit is through the space between domain 1, domain 2 and the active site loop (Figure 11B, Video S3).

**Table 2:** Residue-wise interactions with HMB during its exit in the SMD runs.

Path Residue	C1	F1	F2
R11^b^	49.2	69.5	78.2
Q19^b^	47.5	45.7	66.3
Y22	21.23	-	53.7
S81	2.1	31.5	4.6
K83^a^	55.5	65.6	89.3
D84^a^	52.7	38.9	6.8
V85^a^	48.1	37.1	6.2
R131	26.5	1.5	6.4
N151^a^	30.1	46.6	16.0
R176^b^	92.2	60.0	65.3
Q198	37.5	34.2	14.9

a Residues that interact for more than 30% occupancy of the simulation time.

b Residues that are suggested to be catalytically important.

Interaction of protein residues with HMB moiety during its exit through each path; numbers indicating the % occupancy of interaction.

### (III) Protein relaxation after exit of product

The active site cavity of PBGD enlarges to accommodate the tetrapyrrole product HMB, as seen from SASA and radius of gyration data of the protein (Figure 4C). After suggesting possible path for the product exit, further investigation on protein relaxation was carried, to study if PBGD regains its initial conformation for the next catalytic cycle.

#### Regaining compactness post relaxation

**Figure 12:**
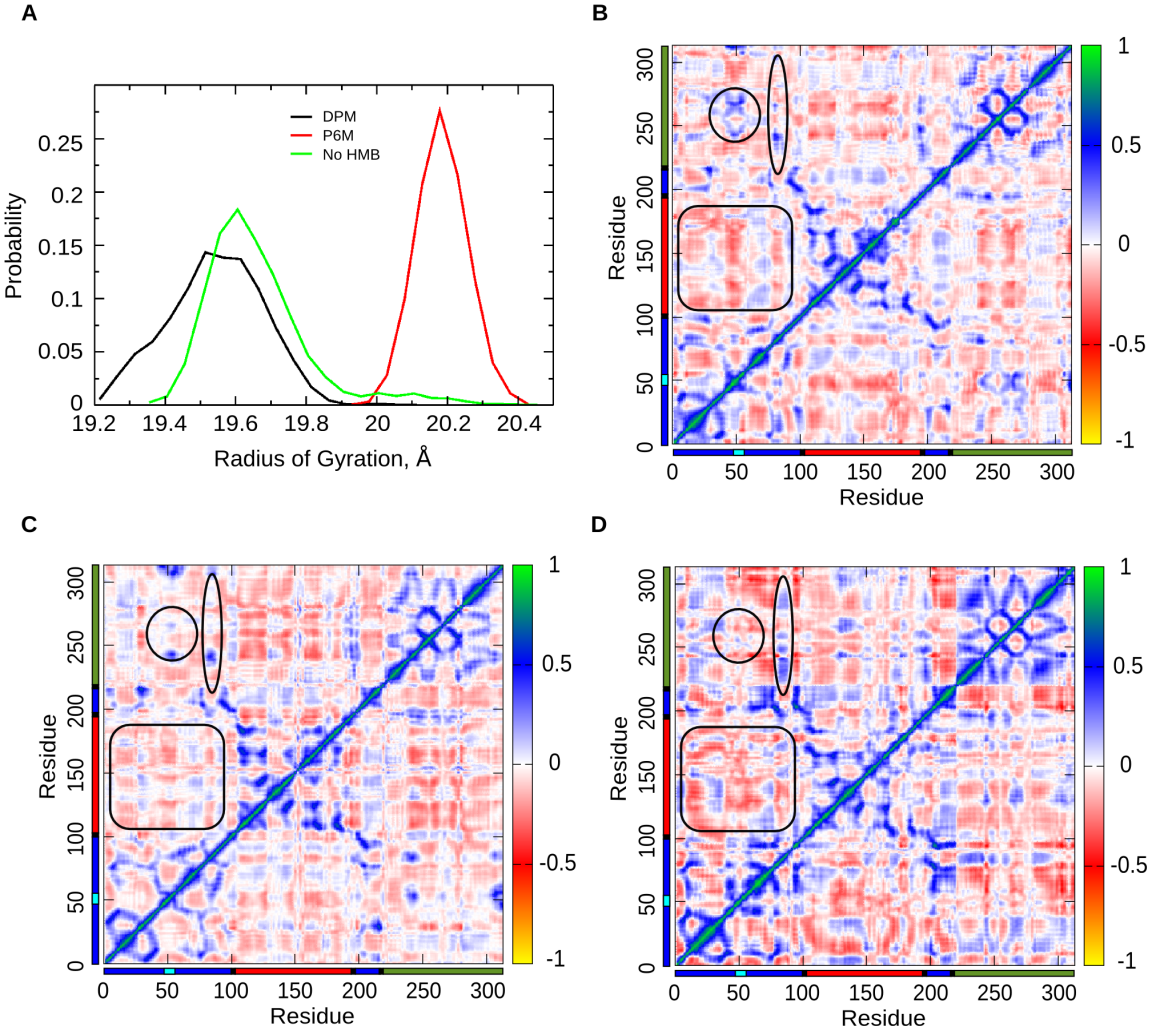
Protein relaxation after product exit. A. Probability distribution graph of radius of gyration of the protein in DPM, P6M and no-HMB stages showing that in the no-HMB stage, the Rgyr falls back close to the DPM stage. DCCM plots of B. no-HMB; C. DPM; and D. P6M stages, showing the differences and similarities in correlation to the DPM stage as the protein relaxes from no-HMB stage. The marked regions in the no-HMB stage resemble more to DPM stage during protein relaxation.

Probability distribution of radius of gyration measured considering the backbone atoms of the protein showed that the value for the protein decreases from 20.2±0.1 Å in P6M stage to 19.6±0.1 Å in the no_HMB stage, which is comparable to that observed during the DPM stage of chain elongation (19.4±0.1 Å) (Figure 12A).

#### Restoration of correlations during relaxation

The DCCM plots of DPM, P6M and no-HMB (for last 50ns of the 150ns trajectory) stages show few significant changes supporting protein relaxation (Figure 12 B, C, D). The region 50–60 and 240–260 that are positively correlated in P6M stage, gradually gets negatively correlated in no-HMB stage to resemble that in the DPM stage. The region 80–100 tends to become more positively correlated to domain 3 as it goes from P6M to no-HMB stage similar to that in the DPM stage. Also, domain 2 region, which is more negatively correlated to domain 1 in P6M stage gradually, gets few positive correlation regions as it proceeds to no-HMB stage resembling the DPM stage.

#### Structural changes indicating relaxation

**Figure 13:**
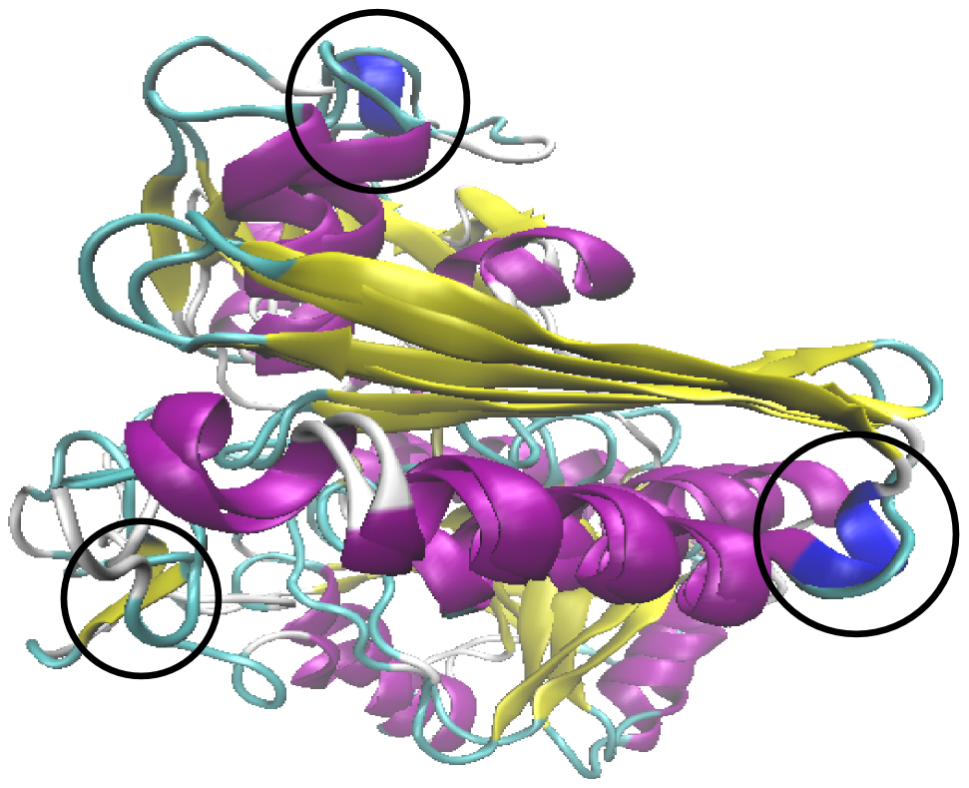
Comparison of average structures of DPM and no-HMB stages. Superposed average structures of the DPM and no-HMB stages. The encircled regions show secondary structures that are yet to be regained after the protein relaxation for 150 ns.

A comparison of the average structures of DPM and no-HMB stages showed significant conformational similarities between the two stages with a RMSD of 3.75 Å. Secondary structural comparison of the two structures showed (Figure 13) that most of the secondary structural elements are regained during the protein relaxation, except for a few retentions. The beta bridge formed between the active site loop and C-terminal is retained. A turn in 115–119 region and a turn of helix in domain 3 are not regained.

From the analyses, it is clear that the protein relaxes to regain its initial conformational state and residue-wise correlations, indicating ability of the protein to resume the next catalytic cycle.

## Discussion

All the hypotheses and speculations that have been proposed on the catalytic mechanism of PBGD prior to this study were based on the structures of the DPM stage alone [5] [8] [13] [14], as the structures of subsequent catalytic stages of PBGD are unknown. Therefore, it was imperative to use computational studies to understand the protein dynamics through all the stages of catalysis. MD studies on PBGD helped in gaining important insights about its structural changes as it catalyzes the formation of 1-hydroxymethylbilane using four units of porphobilinogen, which includes polypyrrole elongation, exit of the product and relaxation of the protein. The flexibility of the active site loop is of significance in modulating step-wise substrate binding. Yan et al., have made similar observations in their NMR study of the bisubstrate enzyme 6-hydroxymethyl-7,8-dihydropterin pyrophosphokinase (HPPK) [28]. The active site loop dynamics and the domain movements appear to be important in the chain elongation process in PBGD. Conserved residues D50, K55 and R149 modulate the dynamics of the active site loop. Most of the conserved arginine residues in the active site contribute in stabilizing the polypyrrole chain through salt-bridge interactions with its acetate and propionate side chains. Radius of gyration and SASA data indicated a decrease in the compactness of the protein during chain elongation.

There have been some speculations on the domain movements in PBGD during the polypyrrole accommodation, based on the dynamics of proteins with similar topology [29], [30]. Gerstein et al., studied Lactoferrins that have two domains similar to those in PBGD. They observed a see-saw movement in the hinge region between the lactoferrin domains [29]. Therefore, it was speculated that domains 1 and 2 in PBGD may exhibit similar dynamics. Using Gaussian network models, Keskin et al., studied the dynamics of a few substrate binding proteins that have domain architecture resembling the Rossmann fold. They suggested domains 1 and 2 in PBGD could be involved in ‘cooperative opposite direction fluctuations’ about a hinge region similar to lysine/arginine/ornithine-binding protein (LAO) dynamics [30]. Louie et al., studied the EcPBGD crystal structure and suggested that domains 1 and 2 might twist open about the hinge between them to increase the volume of active site cleft, with domain 3 moving away from the two domains [16]. Song et al., studied the hPBGD crystal structure and suggested similar movements of the domains 1 and 2 [13]. Domain motions, suggested by Roberts et al., for AtPBGD, state that domain 2 and domain 3 probably move in a concerted manner relative to domain 1, like a ratchet handle [14]. This movement might have a pulling effect on the growing pyrrole chain. Detailed dynamics of the PBGD protein was never studied before. From our observations of the different stages of the protein through essential molecular dynamics, it can be hypothesized that domain 1 and domain 2 move apart about the hinge region to create space for the growing pyrrole chain, while the cofactor turn is inclined towards domain 2.

The accommodation of the polypyrrole within the active site is another question addressed in this study. Roberts et al., hypothesized that the C1 ring (A ring) of the polypyrrole moves from its position in a way that the C2 ring (B ring) could occupy the C1 position allowing the incoming PBG to occupy the vacated C2 position for catalysis [14]. However, in our MD study, it is observed that the C1 ring of the polypyrrole does not change its position, as mentioned by Roberts et al, to vacate space for the incoming PBGs. Rather, the polypyrrole adjusts itself getting inclined towards domain 2 within the active site such that its terminal ring is exposed to active site residues that have a role in the catalytic mechanism of tetrapolymerization of PBG (Figure S1).

The mechanism by which the final product (HMB) is formed by the hydrolysis of the hexapyrrole has not been discussed in the literature. Based on our simulations, it is observed that the C and D rings of the hexapyrrole are exposed to the solvent. Hence, we speculate that the solvent accessibility to the methyl linker between the B and C rings may facilitate hydrolysis, if it is mediated by the solvent.

After the tetrapyrrole product is cleaved, it has to exit the active site of the protein in order to bind to the next enzyme in the heme biosynthetic pathway. The most probable exit path for the product, HMB, has been proposed using steered molecular dynamics. Choutko et al., have used MD simulations to study the exit of product from chorismate mutase and proposed a favorable path for product exit based on minimal structural rearrangements [31]. Similar criteria, based on expulsion force profile, accessibilities between paths from the active site and minimal structural changes were used by us to predict the probable exit path for the product in PBGD. The proposed path for the exit of HMB is through the space between domain 1, domain 2 and the active site loop. Active site residues R11, Q19 and R176, which play a role in protein catalytic mechanism [2], [18], are also observed to be involved in the exit of the product. Choutko et al., also proposed that residues crucial for catalysis have a possible role in the exit of the product similar to our observation [31].

The exit of product from its catalytic site should cause the distended protein to relax to its initial stage structure. In this study, a 150 ns long MD simulation was performed to observe the relaxation in PBGD after catalysis and product exit. It was evident from the radius of gyration computations that the protein regains the compactness, which was lost in the process of accommodating the tetrapyrrole chain, upon exit of the product. The residue-wise correlations were also seen to regain similar trend as in the DPM stage, thus showing potential of PBGD to resume its next catalytic cycle.

### Conclusion

Molecular dynamics study of the enzyme porphobilinogen deaminase helped in gaining important insights about its structural changes as it catalyzes the formation of the product 1-hydroxymethylbilane using four units of porphobilinogen. The study of the chain elongation process revealed the importance of the active site loop and the domain movements in accommodating the polypyrrole chain. The domains 1 and 2 move apart and the cofactor turn moves towards domain 2 to accommodate the growing pyrrole chain in the active site cleft. Conserved residues D50, K55 and R149 modulate the dynamics of the active site loop, while R11, D84 and R176 play a role in the catalytic mechanism corroborating previous biochemical studies [2], [18]. Steered molecular dynamics employed to study the exit of the product, HMB, helped to propose the most probable exit path. Based on expulsion force profile and minimal structural changes, the proposed path for the exit of HMB is through the space formed between domain 1, domain 2 and the active site loop. Active site residues R11, Q19 and R176, reported as catalytically important, are also involved in the exit of the product. The distended PBGD relaxes gradually upon exit of the product to its initial stage structure to resume its catalytic cycle. The questions of how the substrate PBG diffuses into the active-site and the process by which it is covalently linked to the terminal ring of the cofactor to synthesize the tetrapyrrole product are yet to be answered.

## Methods

### Preparation of starting structures

The EcPBGD structure, 2YPN [11], was used to study the protein dynamics through the stages of chain elongation, exit of product and subsequent relaxation. Modeller 9v8 [32] was used to loop-model the missing residues, 43–59, of the active site loop region. The different stages of pyrrole chain elongation (Fig.1) that were studied in this work are: DPM (PBGD with the DPM cofactor), P3M (PBGD after the first catalytic addition of PBG to DPM, i.e., with P3M moiety), P4M (PBGD with a tetrapyrrole (P4M) moiety), P5M (PBGD with a pentapyrrole (P5M) moiety) and P6M (PBGD with a hexapyrrole (P6M) moiety). The starting structure of the protein for each stage was prepared by docking and covalently attaching a moiety of methylene pyrrolenine (MePy), the substrate intermediate, to the polypyrrole cofactor in the active site cavity of the protein followed by its energy minimization.

#### Validation of loop conformation

For validation the 2YPN active site loop modeled by Modeller9v8, AtPBGD structure (4HTG) [14] was used to model this loop. Dynamics study of the new EcPBGD structure was done for first stage of simulation (DPM). And the results obtained were similar, confirming that the active site loop conformation in 2YPN loop modeled structure, used in this study, was appropriate.

### Pyrrole chain elongation

Explicit solvent molecular dynamics simulations of the different stages of PBGD were performed using Gromacs 4.5.5 [33] with G53a6 united-atoms force field [34]. Force field parameters for the covalently attached cofactor, DPM and the subsequent chain extensions, P3M, P4M, P5M and P6M, were obtained from ATB server [35]. The systems were solvated in an octahedron box with a 9 Å layer of SPC/E water model [36]; protein charges were neutralized by adding sodium ions. The systems were then energy minimized using steepest descent method till convergence was reached or for 7000 cycles. NVT and NPT position restrained equilibrations were done for 200 ps and 1 ns respectively with V-rescale temperature coupling [37] and Parrinello-Rahman pressure coupling [38] for the protein and non-protein parts separately. The temperature was gradually raised from 0 K to 300 K at 3 K/ps. Bond lengths were constrained using LINCS algorithm [39]. Periodic boundary conditions were employed to minimize edge effects and the electrostatic computations were done using Particle Mesh Ewald [40], [41] with interpolation order of 4, tolerance of 1e-5 and fourier spacing of 1.6 Å. The DPM and P3M stages were simulated for 35 ns each. The addition of an extra pyrrole ring to P3M resulted in the domains 1 and 2 moving far apart. In order to prevent this, harmonic distance restraints were applied to the centers of mass of the domains 1 and 2 for 15 ns, followed by unrestrained dynamics of 35 ns in each of the subsequent stages (P4M, P5M and P6M stages). The unrestrained simulation trajectories were used for analysis.

### Exit mechanism

After the P6M stage, the hexapyrrole was cleaved at the bridging carbon atom between B and C rings of the moiety, and an -OH group was attached to the linker carbon atom forming the tetrapyrrole product, 1-hydroxymethylbilane, leaving DPM cofactor attached to PBGD (HMB stage). This set up was simulated for 60 ns.

The possible channels for the exit of HMB from PBGD were identified using a pymol plugin CAVER [42]. The structure of PBGD after the HMB stage was used as input for CAVER; center of mass of HMB coordinates were taken as starting point for detecting channels. CAVER predicted three possible exit channels, two (F1 & F2 channel) near the F ring and the third (C1 channel) close to the C ring of HMB (Figure 13).

The PBGD structure from the HMB stage was used as the starting structure to carry out the SMD simulations. Trial SMD runs at variable pulling rates (50 Å ns^−1^, 5 Å ns^−1^) were done to pull HMB from PBGD in each of the 3 directions. The center of mass of protein is used as the reference group and the pull force is applied to the center of mass of either the C ring of HMB moiety in the C1 direction or the F ring of HMB moiety in the F1 and F2 directions at a constant rate of 1 Å ns^−1^. A force constant of 1000 kJ mol^−1^ nm^−2^ was used for the pulling experiments.

### Protein relaxation

The ligand (HMB) was then removed after simulating the HMB stage for 60 ns. The system in the absence of HMB was simulated for 150 ns to observe the protein relaxation process (no-HMB stage).

### Trajectory and structural analyses

Trajectories were analyzed using VMD 1.9.1 [43] and Gromacs 4.5.5. VMD was used to calculate the hydrogen bond interactions and the root mean square deviations (RMSDs) of the backbone atoms of the protein for all the stages of pyrrole chain elongation. The loop modeled and energy minimized 2YPN structure was used as the reference for RMSD calculations. DisRg, a VMD plugin was used for calculation of the radius of gyration (Rgyr). Gromacs was used to calculate the root mean square fluctuations (RMSFs) of the Cα atoms and the solvent accessible surface area (SASA) of the entire protein. Combined essential dynamics [44] and dynamic cross-correlation matrix (DCCM) analysis [45] were performed on the concatenated trajectory of all the stages of pyrrole chain elongation, fitted to the same reference structure, to analyze the cumulative effect of domain motions. Principal component analysis (PCA) was performed using NMWiz, a VMD plugin [46].

Interactions among residues and with the cofactor were studied by calculating the minimum distance between possible donor and acceptor atoms of the respective residues and each ring of the polypyrrole chain. A residue is considered to be interacting with the cofactor if its distance with any polar atom of pyrrole ring is less than 3.5 Å. Based on this criterion, persistence of such interactions in the trajectory were calculated. Distance between domains 1 and 2 was computed using the center of mass of interface residues from domain 1 (residues 14 to 19) and domain 2 (residues 150 to 152 and 173 to 177), to track the domain movements. Volume of the active site cavity along each trajectory was calculated using POVME [47].

## Supporting Information

Figure S1 **Interactions of the active site residues with the growing pyrrole chain.** Stereograms of the average structure showing the interaction of growing pyrrole chain during the stages of chain elongation A. DPM; B. P3M; C. P4M; D. P5M; E.P6M.
(TIF)

Figure S2 **Possible channels of exit for HMB from PBGD predicted using CAVER (a Pymol plugin).** Structure of PBGD showing the 3 possible channels (C1, F1 and F2) for the exit of HMB from PBGD detected by CAVER.
(TIF)

Video S1 **Principal component 1.** Domain 1 and domain 2 are negatively correlated with each other. Cofactor turn moves towards domain 2. Same domain coloring is used in all videos.
(MPG)

Video S2 **Principal component 2.** Active site loop is negatively correlated to domain 2.
(MPG)

Video S3 **Exit mechanism of HMB from PBGD.** HMB exits through the space between domain 1, domain 2 and active site loop. R11, Q19 and R176 interacts with HMB depicting their role in product exit. HMB in van der Waal representation and R11, Q19 and R176 in stick representation.
(MPG)
