# Supplementary figures and images for: Structural Insights into E. coli Porphobilinogen Deaminase during Synthesis and Exit of 1-Hydroxymethylbilane
Source: PLoS Comput Biol. 2014 Mar 6;10(3):e1003484. doi: 10.1371/journal.pcbi.1003484 (PMC3945110; doi:10.1371/journal.pcbi.1003484)

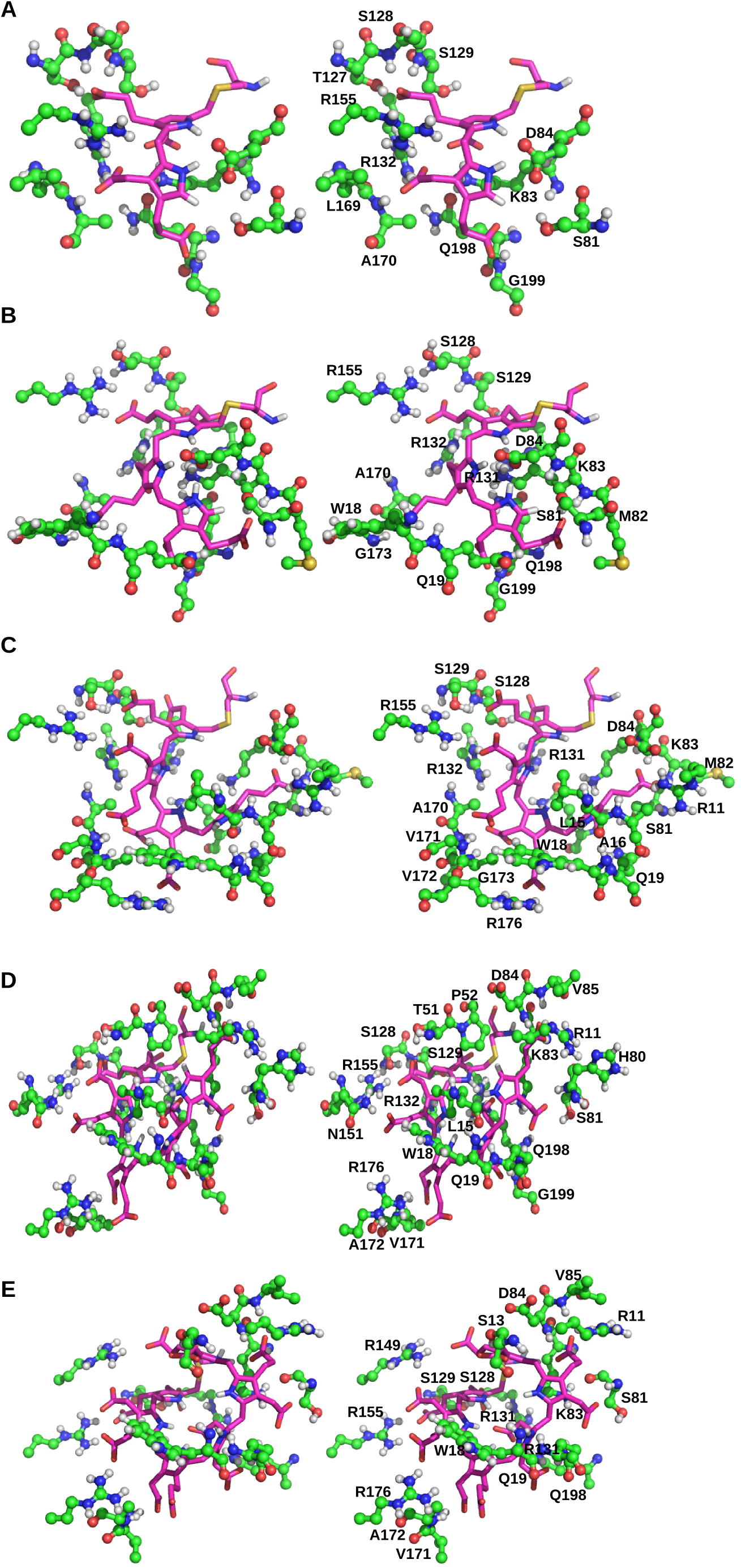

Supplement: Figure S1 — Interactions of the active site residues with the growing pyrrole chain. Stereograms of the average structure showing the interaction of growing pyrrole chain during the stages of chain elongation A. DPM; B. P3M; C. P4M; D. P5M; E.P6M. (TIF) [file pcbi.1003484.s001.tif]

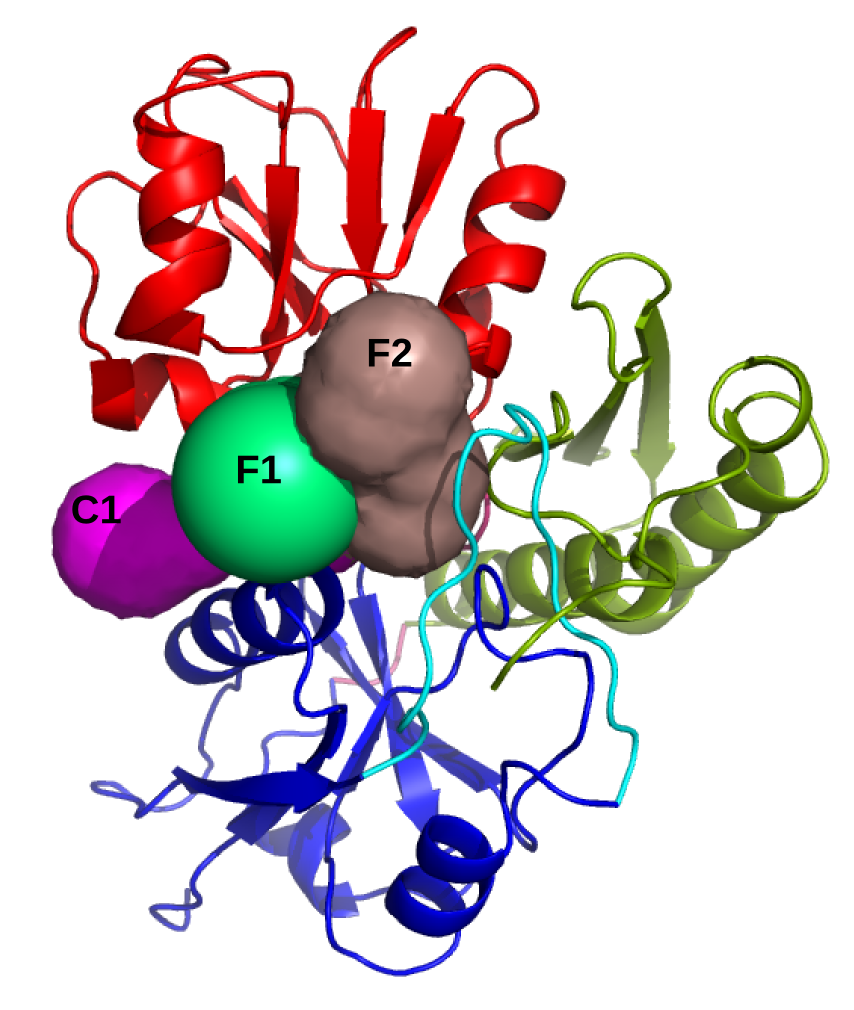

Supplement: Figure S2 — Possible channels of exit for HMB from PBGD predicted using CAVER (a Pymol plugin). Structure of PBGD showing the 3 possible channels (C1, F1 and F2) for the exit of HMB from PBGD detected by CAVER. (TIF) [file pcbi.1003484.s002.tif]
